# Gould’s New Medical Dictionary

**Published:** 1895-03

**Authors:** 


### Gould's New Medical Dictionary.

The *Medical and Surgical Reporter* of Philadephia ends the forty-second year of its consecutive publication with its issue of December 29th. The chief editorial in this number is devoted to a review of the advances made in Medicine and Surgery during 1894. In speaking of medical literature the editors say:

"'Of the making of books there is no end,' thanks to the prevision of generous publishers, and the literary crop of the year contains some wheat among the tares. A few works have been issued in special lines whose value justifies their existence. For pronounced excellence, general utility, and widest application, Dr. Gould's new medical dictionary deserves specific mention among the publications of 1894."

